# Accuracy and Effects of Clinical Decision Support Systems Integrated With BMJ Best Practice–Aided Diagnosis: Interrupted Time Series Study

**DOI:** 10.2196/16912

**Published:** 2020-01-20

**Authors:** Liyuan Tao, Chen Zhang, Lin Zeng, Shengrong Zhu, Nan Li, Wei Li, Hua Zhang, Yiming Zhao, Siyan Zhan, Hong Ji

**Affiliations:** 1 Research Center of Clinical Epidemiology Peking University Third Hospital Beijing China; 2 Information Management and Big Data Center Peking University Third Hospital Beijing China; 3 Department of Epidemiology and Biostatistics School of Public Health, Peking University Beijing China

**Keywords:** BMJ Best Practice, artificial intelligence, clinical decision support systems, aided diagnosis, accuracy and effect

## Abstract

**Background:**

Clinical decision support systems (CDSS) are an integral component of health information technologies and can assist disease interpretation, diagnosis, treatment, and prognosis. However, the utility of CDSS in the clinic remains controversial.

**Objective:**

The aim is to assess the effects of CDSS integrated with British Medical Journal (BMJ) Best Practice–aided diagnosis in real-world research.

**Methods:**

This was a retrospective, longitudinal observational study using routinely collected clinical diagnosis data from electronic medical records. A total of 34,113 hospitalized patient records were successively selected from December 2016 to February 2019 in six clinical departments. The diagnostic accuracy of the CDSS was verified before its implementation. A self-controlled comparison was then applied to detect the effects of CDSS implementation. Multivariable logistic regression and single-group interrupted time series analysis were used to explore the effects of CDSS. The sensitivity analysis was conducted using the subgroup data from January 2018 to February 2019.

**Results:**

The total accuracy rates of the recommended diagnosis from CDSS were 75.46% in the first-rank diagnosis, 83.94% in the top-2 diagnosis, and 87.53% in the top-3 diagnosis in the data before CDSS implementation. Higher consistency was observed between admission and discharge diagnoses, shorter confirmed diagnosis times, and shorter hospitalization days after the CDSS implementation (all *P*<.001). Multivariable logistic regression analysis showed that the consistency rates after CDSS implementation (OR 1.078, 95% CI 1.015-1.144) and the proportion of hospitalization time 7 days or less (OR 1.688, 95% CI 1.592-1.789) both increased. The interrupted time series analysis showed that the consistency rates significantly increased by 6.722% (95% CI 2.433%-11.012%, *P*=.002) after CDSS implementation. The proportion of hospitalization time 7 days or less significantly increased by 7.837% (95% CI 1.798%-13.876%, *P*=.01). Similar results were obtained in the subgroup analysis.

**Conclusions:**

The CDSS integrated with BMJ Best Practice improved the accuracy of clinicians’ diagnoses. Shorter confirmed diagnosis times and hospitalization days were also found to be associated with CDSS implementation in retrospective real-world studies. These findings highlight the utility of artificial intelligence-based CDSS to improve diagnosis efficiency, but these results require confirmation in future randomized controlled trials.

## Introduction

Rapid and accurate diagnosis is important for inpatients and improves their treatment efficiency and length of hospital stay. Artificial intelligence (AI) techniques are useful in a wide variety of medical and clinical diagnostic systems, including pathological diagnosis [1], ophthalmologic disease [2], radiology [3], and dermatology [4]. AI systems in health care have also focused on acquiring knowledge from nonstandardized databases, such as text [5,6] (using natural language processing) or large structured datasets [7] (using machine learning methods). In recent years, AI has been used in medical research and improved many aspects of medical health. Commonly applied AI techniques include deep neural networks, fuzzy logic, decision trees, Bayesian classifiers, genetic algorithms, and hybrid systems [7-11]. In addition, the causality and explainability of AI are attracting more attention in medicine [12,13].

Many clinical decision support systems (CDSS) have emerged from earlier work in AI and expert systems to gather and represent knowledge that can be simulated for human reasoning and advice [11]. As an integral component of health information technologies, CDSS can assist with disease interpretation, diagnosis, treatment, and prognosis. CDSS have been used for more than 50 years [14]; many have commented on its positive impact on diagnostic quality and patient safety [15-18] and ability to promote optimal treatments [19] and avoid medical errors [20,21]. However, some studies [22-24] have reported a lack of benefits for CDSS and highlight the ability of CDSS to introduce new errors. CDSS have been empirically divided into knowledge-driven and data-driven support systems, and AI-based CDSS have broader application prospects with the accumulation of various data.

As for any health care innovation, CDSS must be rigorously evaluated before their widespread dissemination into clinical practice. Accordingly, we performed a real-world retrospective study to evaluate the effects of a self-developed AI-based CDSS from a modernized and comprehensive hospital in China. The AI-based CDSS was integrated with British Medical Journal (BMJ) Best Practice; the AI tools helped to extract patient information and feed it into different machine learning models and BMJ Best Practice. The initial goal was to assess the levels of agreement regarding patients’ diagnoses between CDSS integrated with BMJ Best Practice and resident doctors. The second goal was to understand whether CDSS integrated with BMJ Best Practice improves the accuracy of admission diagnosis for inpatients and to explore the benefits of CDSS integrated with BMJ Best Practice on the length of patients’ hospital stays.

## Methods

### Study Design and Patient Population

This was a retrospective, real-world observational study using continuously collected data from hospitalized patients across six departments of the Peking University Third Hospital from October 1, 2016, to February 30, 2019. The AI-based CDSS was implemented in the electronic medical record (EMR) on November 1, 2018. In the first part, the diagnostic accuracy of CDSS was verified in the hospitalization records data before CDSS implementation. In the second part, a self-controlled study design was applied to detect the effect of CDSS implementation. We compared data before and after AI-based CDSS implementation.

The study subjects were consecutive patients from the six departments: otolaryngology, orthopedic medicine, respiratory medicine, general surgery, cardiology, and hematology. We used no specific inclusion criteria. Subjects were excluded if missing information for key variables, including admission diagnosis, discharge diagnosis, and the length of hospitalization time in their nonstandardized medical records. The study was approved by the Medical Science Research Ethics Committee of Peking University Third Hospital (serial number: IRB00006761-M2019219). Informed consent from the patients was exempt due to the retrospective nature of the study.

### CDSS-Aided Diagnosis

The AI-based CDSS is a multimodel decision system that integrates rule engines and deep learning based on natural language processing, machine learning, and other technologies. The CDSS was created through the learning of nearly 10 years of real historical cases from the Peking University Third Hospital and combining these data with BMJ Best Practice [25]. BMJ Best Practice provides the latest evidence-based information for diagnosis, prognosis, treatment, and prevention; it is updated daily using robust evidence-based methodologies and real expert opinions.

**Figure 1:**
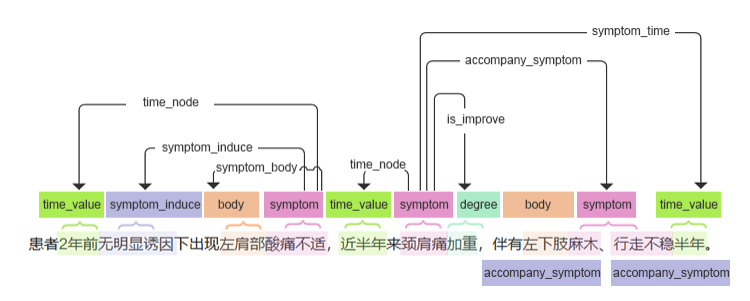
Clinical information extraction based on a bidirectional recurrent neural network.

Based on the medical lexicon built by the medical expert team, natural language processing technology was used to classify the Chinese EMRs. The extracted information was stored in the NoSQL database according to the predefined model structure to provide high-quality structured data to train the diagnostic model. As shown in Figure 1, various structured information could be extracted from historical illnesses, including the symptoms, symptom duration, symptom location, symptom inducers, negative symptoms, and treatment status. The extracted information was fed into different machine learning models and BMJ Best Practice. Based on the patient’s chief concern, history, examination, and test reports, the CDSS recommended a list of possible diagnoses to assist doctors with their diagnoses. The application of CDSS in the EMR is shown in Multimedia Appendix 1.

### Outcomes and Data Collection

There were three primary outcomes: (1) the accuracy of the recommended diagnosis, (2) the consistency of admission and discharge diagnoses, and (3) the length of hospitalization time. There was one secondary outcome: the confirmed length of diagnosis time. The accuracy of the recommended diagnosis was used to evaluate the diagnostic accuracy of the CDSS; the other three outcomes were applied to detect the effect of CDSS implementation.

The accuracy of the recommended diagnosis referred to its consistency with the discharge diagnosis of the patient. The CDSS recommended 10 possible diagnoses according to their probability (from large to small) after referral to the BMJ Best Practice. If the first recommended diagnosis was consistent with the patient’s discharge diagnosis, the record was flagged as a first-rank diagnosis. If one of the first two of the 10 recommended diagnoses was consistent with the patient’s discharge diagnosis, the record was flagged as a top-2 diagnosis. If one of the first three of the 10 recommended diagnoses was consistent with the patient’s discharge diagnosis, the record was flagged as a top-3 diagnosis. If 10 of 10 recommended diagnoses were not consistent with the patient’s discharge diagnosis, the record was flagged as “incorrect.” The discharge diagnosis was affected by the recommended diagnosis from the CDSS after CDSS implementation; therefore, the accuracy of the recommended diagnosis was only tested in the data before CDSS implementation.

The consistency of the admission and discharge diagnoses were analyzed in the data before and after the CDSS implementation. When an inpatient was admitted to the hospital, the doctor made a preliminary admission diagnosis based on the patient’s condition (including past medical history, current medications, history and examination of presenting complaint, social history) and their experience. The preliminary admission diagnosis was recorded in the progress notes. After various examinations after admission, doctors revised the preliminary admission diagnosis and eventually produced a discharge diagnosis. The admission diagnosis was affected by the CDSS after CDSS implementation. The length of hospitalization days referred to the number of days from admission to discharge, which was affected by both patient diagnosis and treatment. The confirmed length of diagnosis time (days) was the duration between preliminary admission diagnosis and definite diagnosis.

Data were extracted from the electronic hospital information system, which routinely records patient information. Those data consisted of patient demographic data, diagnostic data, time of admission, discharge data, and the recommended diagnosis provided by the CDSS. As this was a retrospective study, we used patient data that were not provided with explicit consent for research purposes. No sensitive information that allowed the identification of individuals (eg, postcode, area) were transferred to the research team. All individual patient information was deidentified.

### Statistical Analysis

Data are presented as the mean (SD), median (IQR), or number (percentage) as appropriate. We used independent sample *t* tests or the Mann-Whitney *U* test for the comparison of continuous data and the chi-square test for categorical data. Multivariable logistic regression models were used to determine the effect of CDSS on the consistency and hospitalization time (≤7 days), adjusted for patient gender and age. Single-group interrupted time series analysis was performed to assess the effects of CDSS [26-28]. Time series data were analyzed using an interrupted time series analysis model to assess changes in the levels and trends of the consistent rates of admission and discharge diagnosis, and the rate of hospitalization time of 7 days or less before and after CDSS implementation.

For the missing data of confirmed length of diagnosis time (days), only the complete-case analysis was conducted. In view of the long study span (October 1, 2016, to February 30, 2019), subgroup analysis was performed from January 1, 2018, to February 30, 2019. The content of the subgroup analysis was identical to the entire analysis. *P* values of .05 or less for two-tailed analysis were deemed statistically significant. Analyses were performed with Stata 14.0 and R version 3.5.1 (R Foundation for Statistical Computing).

### Patient and Public Involvement

Neither patients nor the public were involved in this study. Findings will be actively disseminated through conference presentations, publications in academic journals, and commentary in news media to promote the popularization and application of CDSS.

## Results

### Data and Patient Characteristics

Data were used from hospitalized patients in six clinical departments from December 2016 to February 2019. There were a total of 34,113 hospital records, including 27,250 (79.88%) before the CDSS was online, and 6863 (20.12%) after the CDSS was online. Of the 34,113 hospital records, 16,044 were from females, accounting for 47.03%. The mean age of patients was 54.77 (SD 18.55) years. There were more males and older patients before the CDSS, and the differences were statistically significant before and after the CDSS (*P*<.001, Table 1).

**Table 1:** Patient record characteristics before and after CDSS (clinical decision support systems) implementation (N=34,113).

Variables		Total	CDSS Online		*P* value
			Before	After	
**Year in hospital, n (%)**					N/A^a^
	2016	5011 (14.69)	5011 (18.39)	0 (0.00)	
	2017	15,106 (44.28)	15,106 (55.43)	0 (0.00)	
	2018	10,752 (31.52)	7133 (26.18)	3619 (52.73)	
	2019	3244 (9.51)	0 (0.00)	3244 (47.27)	
**Department, n (%)**					<.001
	Otolaryngology	5331 (15.63)	4643 (17.04)	688 (10.02)	
	Orthopedic	8042 (23.57)	5634 (20.68)	2408 (35.09)	
	Respiratory medicine	3208 (9.40)	2834 (10.40)	374 (5.45)	
	General surgery	7344 (21.53)	5084 (18.66)	2260 (32.93)	
	Cardiology	6813 (19.97)	5917 (21.71)	896 (13.06)	
	Hematology	3375 (9.89)	3138 (11.52)	237 (3.45)	
**Gender, n (%)**					<.001
	Female	16,044 (47.03)	12,581 (46.17)	3463 (50.46)	
	Male	18,069 (52.97)	14,669 (53.83)	3400 (49.54)	
Age (years), mean (SD)		54.77 (18.55)	55.09 (18.81)	53.53 (17.43)	<.001

^a^N/A: not applicable.

### Verification of the Recommended Diagnostic Accuracy for CDSS

**Figure 2:**
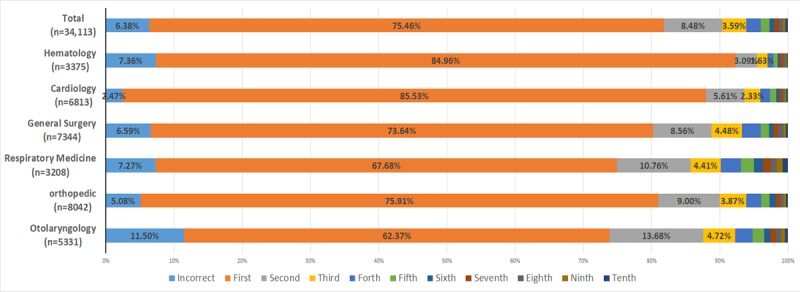
Accuracy of the 10 recommended diagnoses from the CDSS (clinical decision support systems) before implementation in the electronic medical records. “Incorrect” means none of the 10 recommended diagnoses were consistent with the patient’s discharge diagnosis; “first” means the first recommended diagnosis was consistent with the patient’s discharge diagnosis; “second” means the second recommended diagnosis was consistent with the patient’s discharge diagnosis, and so on.

To detect the accuracy of the recommended diagnosis from the CDSS, 27,250 hospitalized records in the EMR were retrospectively assessed before CDSS implementation. The total accuracy rates of the recommended diagnosis by CDSS were 75.46% (20,562/27,250) for first-rank diagnosis, 83.94% (22,873/27,250) for top-2 diagnosis, and 87.53% (23,852/27,250) in top-3 diagnosis. Across departments, first-rank diagnosis accuracy rates varied from 62.37% (2896/4643) to 85.53% (5061/5917), with the highest accuracy rates observed in the cardiology and hematology departments. The incorrect rates were 6.38% in all six clinical departments (Table 2). The accuracy of the recommended diagnosis is shown in Figure 2.

**Table 2:** Accuracy rates of the recommended diagnosis by clinical decision support systems across each department.

Department	Incorrect, n (%)	First, n (%)	First two, n (%)	First three, n (%)
Otolaryngology (n=4643)	534 (11.50)	2896 (62.37)	3531 (76.05)	3750 (80.77)
Orthopedic (n=5634)	286 (5.08)	4277 (75.91)	4784 (84.91)	5002 (88.78)
Respiratory medicine (n=2834)	206 (7.27)	1918 (67.68)	2223 (78.44)	2348 (82.85)
General surgery (n=5084)	335 (6.59)	3744 (73.64)	4179 (82.20)	4407 (86.68)
Cardiology (n=5917)	146 (2.47)	5061 (85.53)	5393 (91.14)	5531 (93.48)
Hematology (n=3138)	231 (7.36)	2666 (84.96)	2763 (88.05)	2814 (89.67)
Total (N=27,250)	1738 (6.38)	20,562 (75.46)	22,873 (83.94)	23,852 (87.53)

### Univariate Comparison Before and After CDSS Implementation

**Figure 3:**
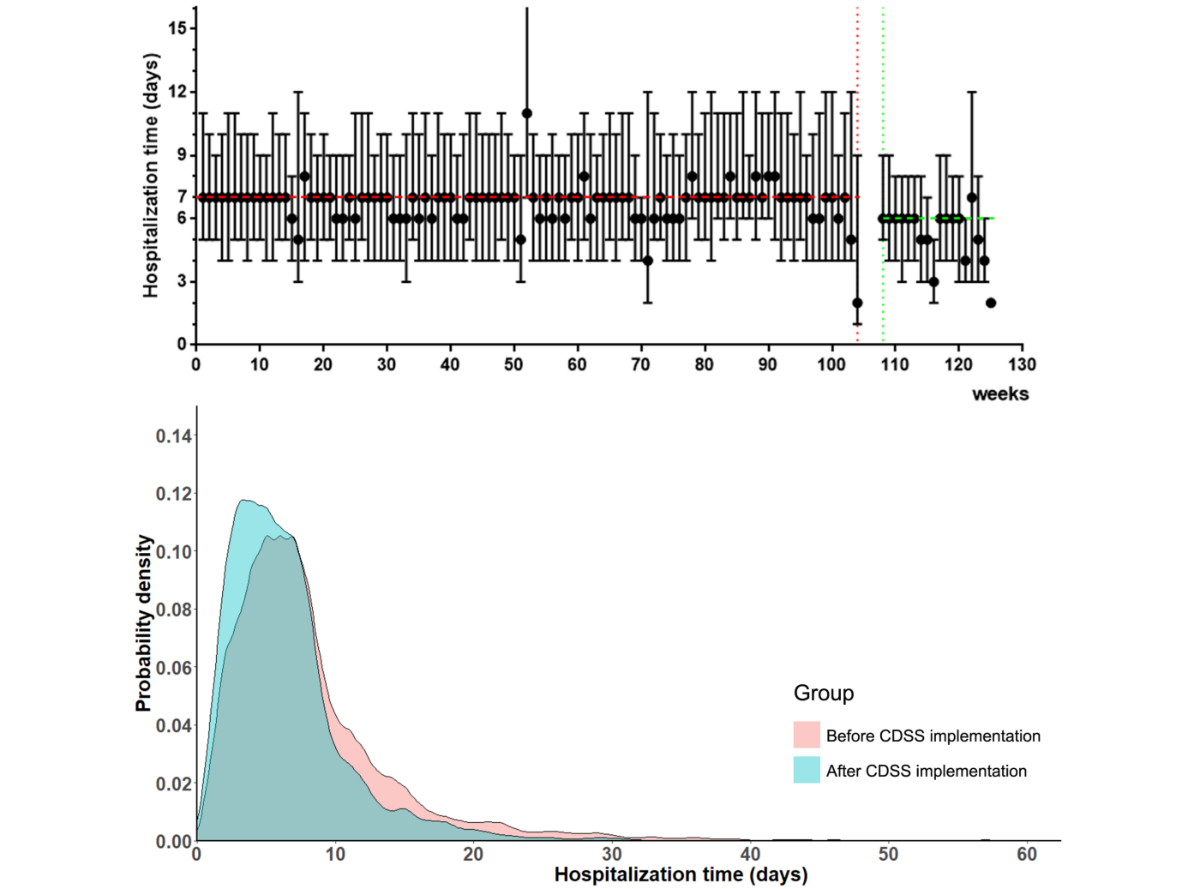
Box plot and probability density diagrams of hospitalization times before and after CDSS (clinical decision support systems) implementation. The red and green dotted lines, respectively, represent the median hospitalization days before and after CDSS implementation; the pink and blue shaded areas, respectively, represent the probability density before and after CDSS implementation.

To explore the effects of the CDSS, the consistency between admission and discharge diagnoses, the length of hospitalization days, and the length of confirmed diagnosis times were compared before and after CDSS implementation. Before the CDSS, the consistency between admission diagnosis and discharge diagnosis was significantly lower than the consistency after CDSS implementation (70.37%, 19,175/27,250 vs 72.64%, 4985/6863, *P*<.001). Median hospitalization days were significantly shortened from 7 (IQR 4-10) to 6 (IQR 3-8) days after CDSS implementation, and the proportion of hospitalization times more than 7 days significantly decreased (*P*<.001). The length of the confirmed diagnosis times also significantly decreased after CDSS implementation (*P*<.001) in 11,912 records that had this information (Table 3). In Figure 3, the box plot and probability density diagram is used to describe the change in hospitalization time before and after CDSS implementation. The line for median hospitalization days was down and the probability density moved to the left after CDSS implementation, suggesting that the average length of hospital stays fell.

In view of the large study span (2016 to 2019), subgroup analysis was performed on the data obtained from 2018 to 2019. The results of the subgroup analysis confirmed that consistency improved after CDSS implementation, while the length of hospitalization and confirmed days were shortened (Multimedia Appendices 2 and 3).

**Table 3:** Comparison of the effects of CDSS (clinical decision support systems) before and after CDSS implementation.

Variables		Total	CDSS Online		*P* value
			Before	After	
**Consistency,^a^ n (%)**					<.001
	Yes	24,160 (70.82)	19,175 (70.37)	4985 (72.64)	
	No	9953 (29.18)	8075 (29.63)	1878 (27.36)	
**Confirmed time (days)^b^**					
	Median (IQR)	1 (0-4)	1 (0-4)	1 (0-3)	<.001
	Mean (SD)	3.10 (5.27)	3.25 (5.48)	2.27 (3.87)	<.001
**Hospitalization time (days)**					
	Median (IQR)	7 (4-9)	7 (4-10)	6 (3-8)	<.001
	Mean (SD)	8.11 (7.55)	8.51 (8.05)	6.49 (4.73)	<.001
**Hospitalization time group (days), n (%)**					<.001
	0-7	20,611 (60.42)	15,774 (57.89)	4837 (70.48)	
	>7	11,476 (39.58)	11,476 (42.11)	2026 (29.52)	

^a^Consistency referred to the consistency between the diagnosis on admission and the diagnosis on discharge.

^b^Only 11,912 records had the length of the confirmed diagnosis times (days), it was the duration between preliminary admission diagnosis and definite diagnosis.

### Multivariable Logistic Regression

We observed a higher consistency between admission and discharge diagnoses and shortened hospitalization days following univariate analysis. To exclude the effect of patient characteristics, multivariable logistic regression analysis was performed. The consistency rates after CDSS implementation increased to 1.078 (95% CI 1.015-1.144) after adjustment for patient gender and age, and the proportion of hospitalization time of 7 days or less increased to 1.688 (95% CI 1.592-1.789) times (Table 4).

In the subgroup analysis, the odds ratio of consistency rates and hospitalization time of 7 days or less were 1.298 (95% CI 1.207-1.397) and 1.757 (95% CI 1.635-1.888), respectively, after CDSS implementation (Multimedia Appendix 4). Males and older patients had higher inconsistency rates and a higher risk of hospitalization time greater than 7 days in all data or subgroup data (Table 4 and Multimedia Appendix 4).

**Table 4:** Multivariable logistic regression analysis of the effects of clinical decision support systems.

Variables		Consistency		Hospitalization time (≤7 days)	
		Adjusted OR (95% CI)	*P* value	Adjusted OR (95% CI)	*P* value
**Group**			0.01		<.001
	Before	1.00		1.00	
	After	1.078 (1.015-1.144)		1.688 (1.592-1.789)	
**Gender**			<.001		<.001
	Female	1.00		1.00	
	Male	0.789 (0.752-0.827)		0.814 (0.778-0.851)	
Age		0.984 (0.983-0.985)	<.001	0.974 (0.973-0.975)	<.001

### Interrupted Time Series Analysis

**Figure 4:**
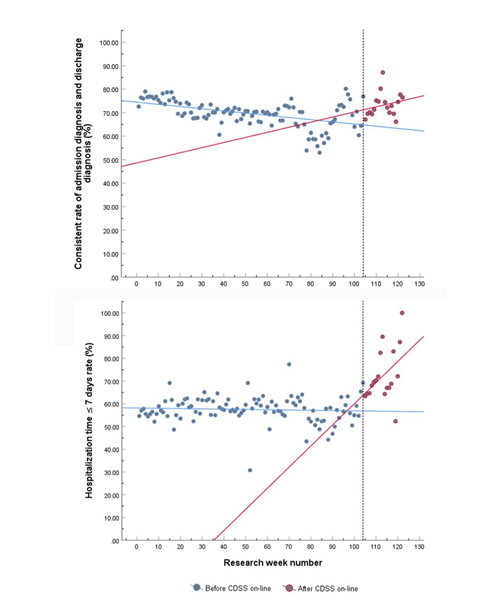
Levels and trend changes of the consistency of admission and discharge diagnoses and the rates of hospitalization time of 7 days or less before and after CDSS (clinical decision support systems) implementation.

As shown in Table 5 and Figure 4, the interrupted time series analysis shows that the levels of change for the weekly consistency rates of admission and discharge diagnoses were 6.722 (95% CI 2.433-11.012) in the level change, indicating that the consistency rates significantly increased by 6.722% after CDSS implementation (*P*=.002). For the proportion of hospitalization times of 7 days or less, a significant increase of 7.837% was observed (95% CI 1.798%-13.876%, *P*=.01) in the level change after CDSS implementation. However, in the subgroup analysis, the level change of the consistency rate was not statistically significant (*P*=.22), but the level change of the proportion of hospitalization times of 7 days or less was statistically significant (*P*=.02) (Multimedia Appendices 5 and 6).

**Table 5:** Estimated levels and trend changes of the consistency rates and hospitalization times of 7 days or less before and after CDSS (clinical decision support systems) implementation.

Outcome variables		Beta (95% CI)	*P* value
**Consistency**			
	Intercept	74.386	
	Before trend	−0.093 (−0.131, −0.055)	<.001
	Level change	6.722 (2.433, 11.012)	.002
	Trend change	0.311 (0.001, 0.620)	.05
**Hospitalization time ≤7 days rate**			
	Intercept	58.146	
	Before trend	−0.013 (−0.047, 0.022)	.47
	Level change	7.837 (1.798, 13.876)	.01
	Trend change	0.941 (−0.032, 1.915)	.06

## Discussion

Large data and digitalization are rapidly expanding in the clinical setting, but health care providers often do not fully exploit these datasets. Clinical decisions are often made by health care professionals during direct patient contact, ward rounds, or multidisciplinary meetings, meaning that decisions are made within seconds to minutes depending on the experience of the health care provider [29]. Computer-based systems can consider all available data, including EMRs, guidelines from evidence-based medicine, and current medical insights. The CDSS contains a vast amount of information that can help clinicians make appropriate decisions for individual patients.

The earliest known CDSS was medication-related and dated back to the 1960s [30]. This system supported pharmacists with drug allergy assessments, dose guidance, drug-drug interactions, and duplicate therapy assessments. These assays were designed using simplistic “if-then-else” logic and did not combine complex algorithms, such as deep neural networks, fuzzy logic, Bayesian classifiers, and hybrid systems. Advanced CDSS were designed to aid clinical decision making using individual patient characteristics and external information to generate health-related recommendations. CDSS were applied for AI [11,31] assessments.

Recent studies have reported the wide application of CDSS combined with AI in clinical settings [3,7,9,11,18,32]. A range of systematic reviews, meta-analyses, or synthesis of systematic reviews have summarized the effects of CDSS in chemotherapy processes [33], cardiovascular risk factors [24], drug allergy checks [34], patient outcomes [15,17], acute care management [35], primary preventive care [36], and chronic disease management [37]. In those studies, CDSS have a positive effect on clinical diagnosis, whereas some have suggested no effect. There are also studies reporting that CDSS poorly presents data and causes alert fatigue to health care providers [38]. Therefore, we designed a retrospective, longitudinal observational study to explore the real-world effect of CDSS-aided diagnoses. The CDSS was self-developed and AI-based, which integrated the optimal BMJ best practices.

BMJ Best Practice is a clinical decision support tool that works at the point-of-care. It offers continually updated, evidence-based, and practical content to all health care professionals [25]. BMJ Best Practice is one of the best clinical decision support tools for health professionals worldwide [39]. Evidence-based clinical decision support resources may offer well-designed clinical pathways and algorithms, which can save busy clinicians’ time and effort in designing clinical pathways. BMJ Best Practice can help doctors and other health care professionals find immediate, current, and evidence-based answers to important clinical questions [40].

There were 34,113 inpatient records involved in this study accumulated from six clinical departments. Of these, 27,250 (79.9%) records were before the CDSS implementation, and the simulations of diagnostic accuracy were performed in them. The total accuracy rates of the recommended diagnosis by AI-based CDSS were 75.46% in first-rank diagnosis, 83.94% in top-2 diagnosis, and 87.53% in top-3 diagnosis. The incorrect rates were 6.38%. The accuracy rates were high, consistent with other studies that have also shown that AI-based tools are accurate in aiding diagnosis. Hannun et al [9] used deep neural networks to detect and classify cardiologist-level arrhythmias in ambulatory electrocardiograms. Their results showed good classification accuracy (area under the curve=0.97). Attia et al [7] tested the application accuracy of AI for electrocardiograms with accuracies of 85.7% observed. Wildman-Tobriner et al [3] showed that an AI-optimized Thyroid Imaging Reporting and Data System (TI-RADS) could modestly improve specificity and maintain sensitivity compared with the American College of Radiology TI-RADS. Similar diagnostic tools based on different AI algorithms had good accuracy for the detection of lymph node metastases in women with breast cancer [1], dermatologist-level classification of skin cancer [4], diabetic retinopathy and diabetic macular edema [41], and multiclass diagnosis of Alzheimer disease [42]. These results suggest that diagnosis systems based on AI have good diagnostic accuracy, but their clinical application requires verification.

In addition to simulation studies, we designed a before-and-after comparison to explore the accuracy of the admission diagnosis after CDSS implementation, with outcomes measured as the consistency between admission and discharge diagnoses. Before CDSS implementation, the admission diagnosis could only be made based on patient information (eg, outpatient examinations) and the doctor’s experience. The patient’s admission diagnosis was assisted by the CDSS recommendation after CDSS implementation. Our results showed that the consistency significantly improved after CDSS implementation in all analyses (from 70.37% to 72.64%, *P*<.001) and subgroup analyses (from 66.59% to 72.64%, *P*<.001), although the increase was not large. Similar results were detected in multivariable logistic regression and interrupted time series analysis, suggesting that the application of CDSS could improve the consistency of admission and discharge diagnoses. Dhombres et al [43] showed that an intelligent scan assistant system for early pregnancy diagnosis by ultrasound could improve the rate of correct diagnosis to 20%. A prospective multicenter study assessed the impact of CDSS to predict progression in patients with subjective cognitive decline and mild cognitive defects [44] and found that the prediction of progression changed in 13% of patients when CDSS was applied. The clinicians’ confidence in their predictions also increased when using CDSS [44].

After CDSS implementation, the confirmed time and hospitalization time were significantly shorter (decrease of 0.98 days and 2.02 days in all data, respectively). We observed a similar trend via subgroup and multivariable analyses. In the interrupted time series analysis, the rates of consistency and hospitalization time of 7 days or less increased by 6.72% and 7.84%, respectively, after CDSS implementation. Although meta-analyses showed that the application of CDSS did not have clear clinical benefits in cardiovascular risk management [24], a positive effect of CDSS has been proposed in other studies [14,43,45]. We similarly confirmed the clinical benefits of CDSS implementation from the perspective of aided diagnosis to improve the accuracy of diagnosis and shorten confirmed diagnosis times and the length of hospitalization time. This study embedded AI-based CDSS into EMRs and evaluated the effect of CDSS on diagnosis in six clinical departments. These results reflect the practical benefits of CDSS in our hospital. However, because only the benefits of CDSS to assist diagnosis were assessed, future studies should evaluate the role of CDSS in assisting treatment decision-making decisions in the real world.

The study had several limitations. First, the multivariate analysis of CDSS did not take into account the impact of the doctor’s personal information, such as education level, technical post, and work experience. Second, the multivariate analysis did not consider the impact of the individual patient’s disease severity. However, because a large sample size was continuously enrolled, a balance in disease severity would be anticipated. Third, this study did not consider the impact of time factors and the adjustments of national basic health policy from 2016 to 2019. To eliminate the influence of time factors, we performed a subgroup analysis on data from 2018 and 2019, and we believe that time factors and health policy changes would have little impact in a relatively short period of time (less than 2 years). Fourth, the amount of data after CDSS application in this study was small, accounting for only 20.1% of the total datasets. Finally, the CDSS application in China should be trained not only by global evidence but also by regional evidence, including traditional Chinese medicine. In addition, the conclusions of the study were limited by the retrospective nature of the cohort; strict randomized controlled trials are needed to explore the accuracy of CDSS in aided diagnosis.

There are many kinds of CDSS, ranging from simple logical judgments to complex AI algorithms, adverse drug reactions to data-driven aided diagnosis and treatment. From these, various forms of CDSS are emerging. Using the current development and application of CDSS, there is no unified standard to restrict use; therefore, further evaluations and training are required before CDSS tools are adopted into clinical practice. Standard guidelines for CDSS classifications and eligibility specifications should also be published to ensure reproducibility. In the future, more complex AI-based CDSS can be implemented into the EMR. We believe that this application can create new horizons for scientific research and improve the quality of health and health care.

## Appendix

Multimedia Appendix 1 Picture of the actual application of Clinical decision support systems (CDSS) in Electronic Medical Record (EMR).

Multimedia Appendix 2 Comparison before and after the Clinical decision support systems (CDSS) in subgroup analysis.

Multimedia Appendix 3 Box-plot and probability density diagram of the hospitalization time in the days before and after Clinical decision support systems (CDSS) implementation in subgroup analysis.

Multimedia Appendix 4 Multivariable logistic regression analysis of the effects of Clinical decision support systems (CDSS) in subgroup analysis.

Multimedia Appendix 5 Estimated levels and trend changes of the consistency rates and hospitalization times ≤ 7days before and after Clinical decision support systems (CDSS) implementation in subgroup analysis.

Multimedia Appendix 6 Levels and trend changes of the consistency of admission and discharge diagnosis and the rates of hospitalization time ≤ 7 days before and after Clinical decision support systems (CDSS) implementation in subgroup analysis.

## Abbreviations

AI: artificial intelligence
BMJ: British Medical Journal
CDSS: clinical decision support systems
EMR: electronic medical record
