# Supplementary material for: Accuracy and Effects of Clinical Decision Support Systems Integrated With BMJ Best Practice–Aided Diagnosis: Interrupted Time Series Study
Source: JMIR Med Inform. 2020 Jan 20;8(1):e16912. doi: 10.2196/16912 (PMC6997922; doi:10.2196/16912)
Supplement: Multimedia Appendix 1 [file medinform_v8i1e16912_app1.docx]

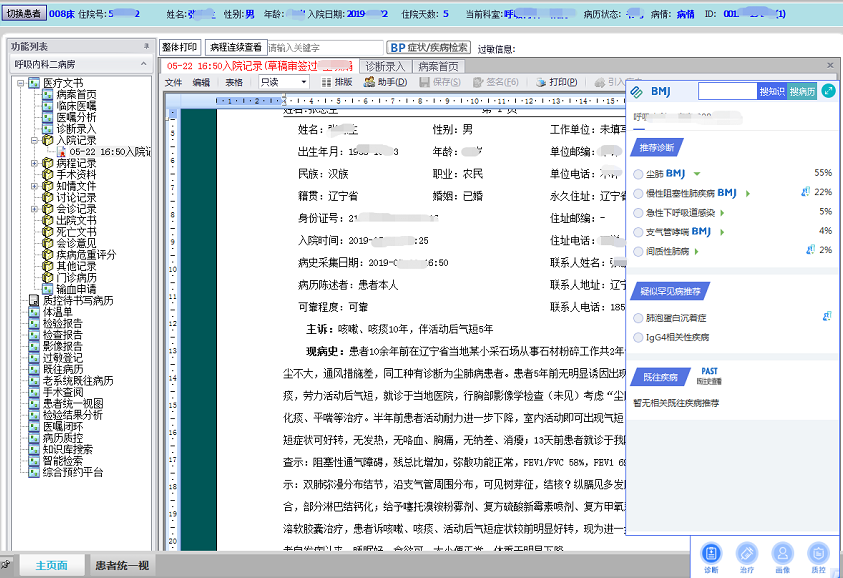


**Figure S1. Picture of the actual application of Clinical decision support systems (CDSS) in Electronic Medical Record (EMR)**
