# Supplementary material for: Accuracy and Effects of Clinical Decision Support Systems Integrated With BMJ Best Practice–Aided Diagnosis: Interrupted Time Series Study
Source: JMIR Med Inform. 2020 Jan 20;8(1):e16912. doi: 10.2196/16912 (PMC6997922; doi:10.2196/16912)
Supplement: Multimedia Appendix 2 [file medinform_v8i1e16912_app2.docx]

**Table S1. Comparison before and after the CDSS in subgroup analysis**

|  | **Total** | **CDSS On-line** | | ***P*** |
| --- | --- | --- | --- | --- |
|  |  | Before | After |  |
| Consistency^a^ |  |  |  | <.001 |
| Yes | 9735(69.56) | 4750(66.59) | 4985(72.64) |  |
| No | 4261(30.44) | 2383(33.41) | 1878(27.36) |  |
| Confirmed time (days) ^b^ |  |  |  |  |
| Median(p25, p75) | 1(0, 3) | 1(0, 3) | 1(0, 3) | .01 |
| Mean(SD) | 2.69±4.72 | 2.93±5.13 | 2.27±3.87 | <.001 |
| Hospitalization time (days) |  |  |  |  |
| Median(p25, p75) | 6(4, 9) | 7(4, 10) | 6(3, 8) | <.001 |
| Mean(SD) | 7.70±7.33 | 8.86±9.01 | 6.49±4.73 | <.001 |
| Hospitalization time group (days) |  |  |  | <.001 |
| 0-7 | 8922(63.75) | 4085(57.26) | 4837(70.47) |  |
| >7 | 5074(36.25) | 3048(42.74) | 2026(29.52) |  |

**Notes:** 13,996 hospital records from January 1st, 2018 to February 30th, 2019 were included in the subgroup analysis; a Consistency referred to the consistency between the diagnosis on admission and the diagnosis on discharge; b Only 5,180 records had the length of the confirmed diagnosis times.
