# Supplementary material for: Accuracy and Effects of Clinical Decision Support Systems Integrated With BMJ Best Practice–Aided Diagnosis: Interrupted Time Series Study
Source: JMIR Med Inform. 2020 Jan 20;8(1):e16912. doi: 10.2196/16912 (PMC6997922; doi:10.2196/16912)
Supplement: Multimedia Appendix 3 [file medinform_v8i1e16912_app3.docx]

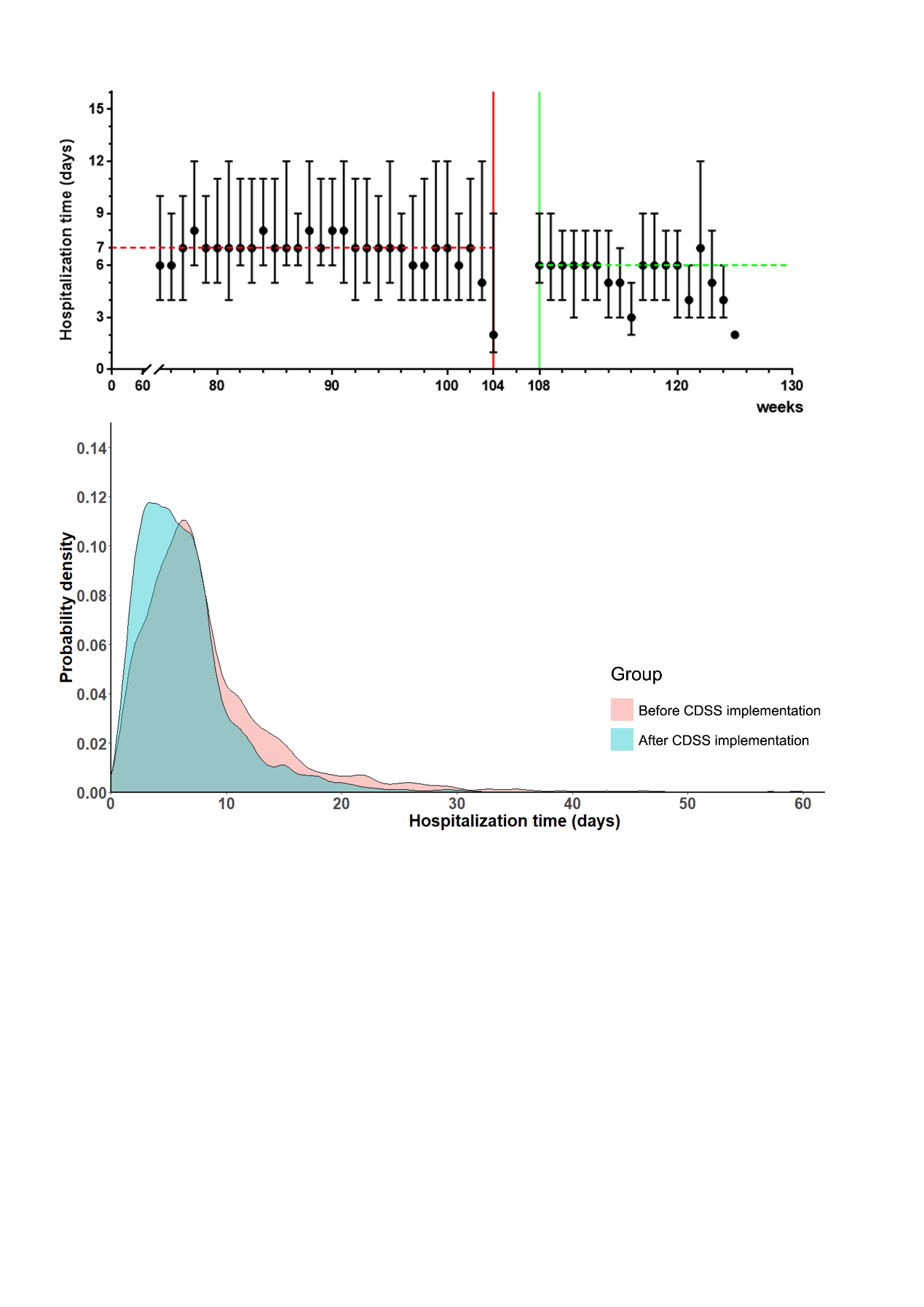


**Figure S2.** Box-plot and probability density diagram of the hospitalization time in the days before and after CDSS implementation in subgroup analysis.

(A: Box-plot, red dotted line represented the median hospitalization days before CDSS implementation, green dotted line represented the median hospitalization days after CDSS implementation; B: Probability density diagram. Pink shadow: before CDSS implementation; blue shadow: after CDSS implementation).
