# Supplementary material for: Accuracy and Effects of Clinical Decision Support Systems Integrated With BMJ Best Practice–Aided Diagnosis: Interrupted Time Series Study
Source: JMIR Med Inform. 2020 Jan 20;8(1):e16912. doi: 10.2196/16912 (PMC6997922; doi:10.2196/16912)
Supplement: Multimedia Appendix 4 [file medinform_v8i1e16912_app4.docx]

**Table S2. Multivariable logistic regression analysis of the effects of CDSS in subgroup analysis**

|  | **Consistency^a^** | |  | **Hospitalization Time (≤7 days)** | |
| --- | --- | --- | --- | --- | --- |
|  | **aOR (95% CI)** | ***P*** |  | **aOR (95% CI)** | ***P*** |
| Group |  | <.001 |  |  | <.001 |
| Before | 1.00 |  |  | 1.00 |  |
| After | 1.298(1.207-1.397) |  |  | 1.757(1.635-1.888) |  |
| Gender |  | <.001 |  |  | <.001 |
| Female | 1.00 |  |  | 1.00 |  |
| Male | 0.835(0.776-0.899) |  |  | 0.840(0.781-0.903) |  |
| Age | 0.985(0.983-0.987) | <.001 |  | 0.973(0.971-0.975) | <.001 |

**Notes:** 13,996 hospital records from January 1st, 2018 to February 30th, 2019 were included in the subgroup analysis; a Consistency referred to the consistency between the diagnosis on admission and the diagnosis on discharge.
