# Supplementary material for: Accuracy and Effects of Clinical Decision Support Systems Integrated With BMJ Best Practice–Aided Diagnosis: Interrupted Time Series Study
Source: JMIR Med Inform. 2020 Jan 20;8(1):e16912. doi: 10.2196/16912 (PMC6997922; doi:10.2196/16912)
Supplement: Multimedia Appendix 5 [file medinform_v8i1e16912_app5.docx]

**Table S3. Estimated levels and trend changes of the consistency rates and hospitalization times ≤ 7days before and after CDSS implementation in subgroup analysis**

| **Outcome variables** |  | **β** | **95% CI** | | ***P*** |
| --- | --- | --- | --- | --- | --- |
| Consistency^a^ | intercept | 65.661 |  |  |  |
|  | before trend | 0.057 | -0.119 | 0.233 | .52 |
|  | level change | 3.459 | -2.106 | 9.023 | .22 |
|  | trend change | 0.160 | -0.202 | 0.522 | .38 |
| Hospitalization Time  ≤7 days rate | intercept | 57.689 |  |  |  |
|  | before trend | -0.042 | -0.233 | 0.150 | .67 |
|  | level change | 8.652 | 1.465 | 15.839 | .02 |
|  | trend change | 0.971 | -0.051 | 1.992 | .06 |

**Notes:** 13,996 hospital records from January 1st, 2018 to February 30th, 2019 were included in the subgroup analysis; a Consistency referred to the consistency between the diagnosis on admission and the diagnosis on discharge.
