# Supplementary material for: Accuracy and Effects of Clinical Decision Support Systems Integrated With BMJ Best Practice–Aided Diagnosis: Interrupted Time Series Study
Source: JMIR Med Inform. 2020 Jan 20;8(1):e16912. doi: 10.2196/16912 (PMC6997922; doi:10.2196/16912)
Supplement: Multimedia Appendix 6 [file medinform_v8i1e16912_app6.docx]

**
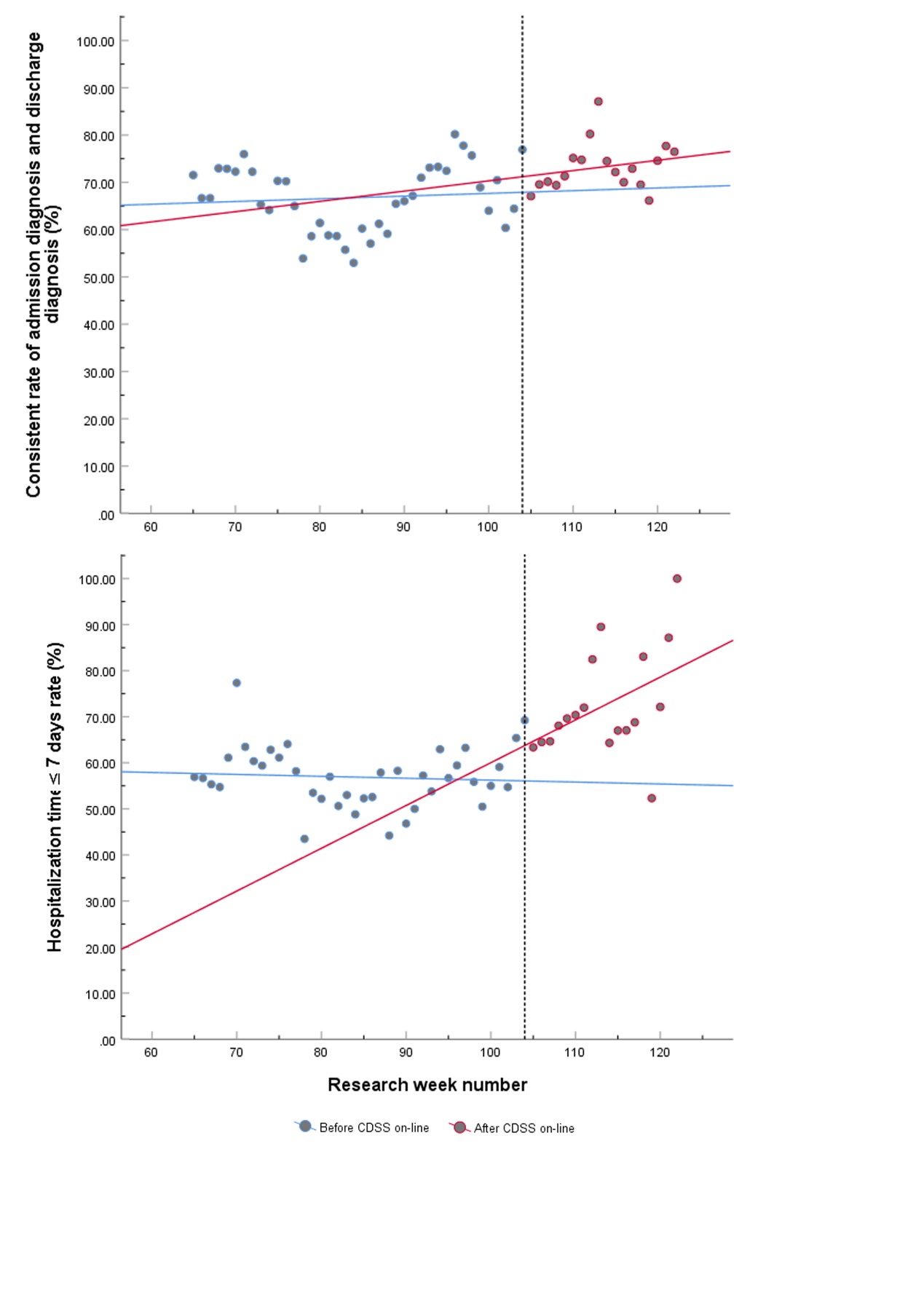
**

**Figure S3.** Levels and trend changes of the consistency of admission and discharge diagnosis and the rates of hospitalization time ≤ 7 days before and after CDSS implementation in subgroup analysis.
